# Curcumin Treatment Ameliorates Hepatic Insulin Resistance Induced by Sub-chronic Oral Exposure to Cadmium LOAEL Dose via NF-κB and Nrf2 Pathways

**DOI:** 10.1007/s12011-024-04314-1

**Published:** 2024-08-06

**Authors:** Victor Enrique Sarmiento-Ortega, Diana Moroni-González, Alfonso Diaz, Eduardo Brambila, Samuel Treviño

**Affiliations:** 1 Laboratory of Chemical-Clinical Investigations, Department of Clinical Chemistry, Chemistry Department, Meritorious Autonomous University of Puebla, 14 Sur. FCQ1, Ciudad Universitaria, 72560 Puebla, C.P Mexico; 2 Department of Pharmacy, Faculty of Chemistry Science, Meritorious Autonomous University of Puebla, 22 South. FCQ9, Ciudad Universitaria, 72560 Puebla, C.P Mexico

**Keywords:** Cadmium, NF-κB, Nrf2, Insulin resistance, Curcumin

## Abstract

Cadmium (Cd) is a global pollutant, and its accumulation in the liver causes oxidative stress, inflammation, insulin resistance (IR), and metabolic complications. This study investigated whether curcumin treatment could alleviate hepatic IR in Wistar rats exposed to sub-chronic cadmium and explored the underlying molecular pathways. Male Wistar rats were divided into a control group (standard normocaloric diet + cadmium-free water) and a cadmium group (standard normocaloric diet + drinking water with 32.5 ppm CdCl_2_) for 30 days. Oral glucose tolerance, insulin response, and IR were assessed using mathematical models. Liver tissue was analyzed for markers of oxidative stress, inflammation, and key regulatory pathways, including NF-κB, Nrf2, MAPKs (JNK and p38), and the IRS1-Akt pathway. We established an effective curcumin dose of 250 mg/kg for 5 days orally. Results demonstrated that after 30 days of exposure, cadmium accumulated in the liver, inducing an oxidative and inflammatory state. This was characterized by increased expression of NF-κB, JNK, and p38, along with diminished Nrf2 expression, hepatic IR, hyperglycemia, and hyperinsulinemia. Curcumin treatment effectively alleviated these metabolic disorders by restoring the balance between NF-κB and Nrf2 in the liver, modulating the MAPK pathway, and, consequently, improving oxidative and inflammatory balance. In conclusion, this study suggests that cadmium induces hepatic IR through an imbalance between NF-κB and Nrf2 signaling pathways. Curcumin treatment appears to improve these pathways, thereby ameliorating hepatic IR.

**Supplementary Information:**

The online version contains supplementary material available at 10.1007/s12011-024-04314-1.

## Introduction

Cadmium (Cd), a heavy metal and global pollutant, is primarily absorbed into organisms through contaminated food or water, with inhalation and smoking additional exposure routes [1, 2]. The risk of developing a disease by Cd exposure is categorized into different levels according to an estimate of daily exposure without adverse effects over a specified exposure time. The categorization depends on the time exposure, concentration or dose, toxicokinetics, and toxicodynamics of the metal, and it is referred to as (a) the lowest observed adverse effect level (LOAEL) and (b) no observed adverse effect level (NOAEL). Both dosages often focus on Cd carcinogenicity [2–4]. However, we previously reported that chronic Cd exposure to LOAEL and NOAEL doses disrupts insulin signaling, lipid concentrations, and glucose homeostasis, leading to metabolic syndrome and diabetes in a time-dependent manner [2, 5, 6].

Although the mechanisms are still not fully elucidated, our workgroup has evidenced that the Islets of Langerhans overproduce and secrete insulin after chronic oral exposure to a cadmium LOAEL dose [3]. Chronic hyperinsulinemia causes insulin resistance (IR) in the liver, muscle, and adipose tissue [6]. In the liver, insulin signaling regulates glycogen synthesis and the *novo* lipogenesis simultaneously through the insulin receptor insulin (IRS)–phosphoinositide 3-kinase (PI3K)–protein kinase B (Akt) pathway, also known as a metabolic arm. Previous results showed chronic Cd exposure to LOAEL and NOAEL doses disrupts this pathway, leading to hepatic IR, low glycogen synthesis, and increased triglyceride production [2, 6–8]. These metabolic changes are associated with insulin mitogenic pathway overactivation via mitogen-activated protein kinases (MAPK), which can be triggered by oxidative stress and inflammation, both of which are potently induced by Cd exposure at LOAEL and NOAEL doses [9–11].

Nuclear factor erythroid 2-related (Nrf2), a master regulator of oxidative stress, induces the expression of various detoxifying and antioxidant genes for cell protection [12]. Meanwhile, the nuclear factor kappa-light-chain-enhancer of activated B cells (NF-κB) regulates genes involved in innate and adaptive immune responses [13]. Oxidative stress induces inflammation and vice versa, but each tissue has a threshold, which conditions their physiological, physiopathological, or pathological response. Cadmium accumulation can modulate the expression of both transcriptional factors, disrupting this equilibrium, potentially overwhelming cellular defenses, and contributing to IR. Thus, this delicate balance is crucial for maintaining cellular health. Due to the tissue threshold for Cd management, the literature regarding the topic is controversial. However, a close link exists between the MAPK pathway, NF-κB, and Nrf2, and little has been investigated regarding hepatic IR [2]. Curcumin, a polyphenol with established antioxidant, anti-inflammatory, and anti-tumor properties, has been shown to mitigate the harmful effects of Cd [14]. Therefore, curcumin has received considerable attention due to its therapeutic properties, and the mechanisms underlying some of its actions have recently been investigated. Given that Cd exposure at LOAEL doses likely induces IR through oxidative stress and inflammation, we aimed to examine whether curcumin treatment could ameliorate hepatic IR in Wistar rats by targeting the same molecular pathways affected by sub-chronic Cd exposure.

## Materials and Methods

### Animals and Treatment

**Fig. 1:**
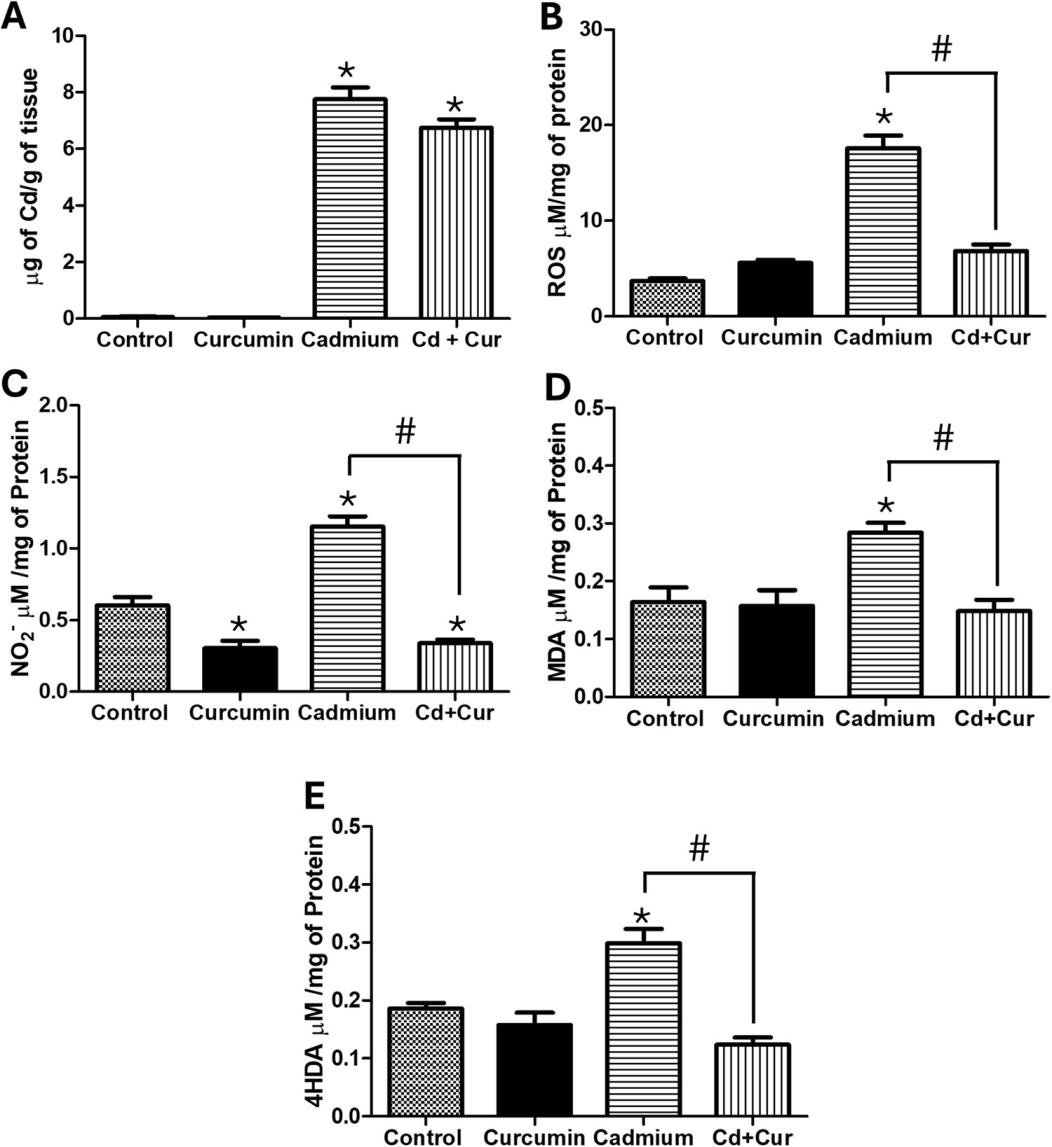
Effect of curcumin administration on hepatic cadmium accumulation and oxidative stress. **A** Cadmium concentration; **B** reactive oxygen species (ROS); **C** nitrites (NO_2_^−^); **D** malondialdehyde (MDA); **E** 4-hydroxyalkenal (4HDA). Results are the mean average of five separate experimental animals per group ± SEM. The symbol “*” indicates a significant difference regarding the control group (*p* ≤ 0.05); the symbol “#” indicates a significant difference regarding the cadmium group (*p* ≤ 0.05), by a one-way ANOVA followed by a Tukey’s post-test

Seventy-five male Wistar rats (2–3 weeks old, 70–80 g) were obtained from the “Claude Bernard” vivarium of the Universidad Autónoma de Puebla. The animals were housed in a controlled temperature environment (22 °C) with a 12-h light/dark cycle and provided with a standard chow diet (5001, LabDiet; St. Louis, MO, USA) and water ad libitum until they reached a weight of 100 g (approximately 1-month-old). Following 1 month of acclimation, the animals were divided into two groups (*n* = 20 each): a control group receiving cadmium-free drinking water (orally) and a cadmium group receiving drinking water containing 32.5 ppm CdCl_2_ (ad libitum) for 30 days. Then, each group was further divided into two subgroups (*n* = 10). One subgroup from each main group received a daily oral gavage of curcumin (250 mg/kg body weight) dissolved in a 100-mM carbonate/bicarbonate buffer (pH 9.6) for 5 days. This dose was chosen based on a pilot study where different curcumin concentrations (0, 50, 100, 250, 500, and 750 mg/kg/day) were administered to rats pre-exposed to cadmium (details in Fig. 1S). The 250 mg/kg dose was selected because it demonstrated the most significant reduction in ROS levels and improvement in hepatic IR. The procedures described in this study were performed following the Standards for the Use and Care of Laboratory Animals described by the Mexican Council for Animal Care (NOM-062-ZOO-1999), as well as the Guide of the National Institutes of Health for the Care and Use of Laboratory Animals and the Ethics Committee of the Autonomous University of Puebla. All applicable international, national, and institutional guidelines for the care and use of animals were followed to minimize possible discomfort.

### Biochemical Assays

After 5 days of curcumin administration, an oral glucose tolerance test (OGTT; 1.75 g/kg) was performed. Food and water were suspended for 4–5 h before collection, and a fasting blood sample (time 0 min) was obtained. After glucose administration, blood samples were taken at 30, 60, and 90 min from the tail vein. Samples were centrifuged at 400 × g for 10 min; the serum was separated and frozen at − 70 °C until analysis. Area under the curve (AUC) was calculated using glucose concentration (BioSystems, Guadalajara, Mexico) and insulin concentration (Diagnostica Internacional Company, Guadalajara, Mexico) data. The homeostatic model assessment insulin resistance (HOMA-IR) and the liver insulin resistance index (LIRI) were determined as we previously reported [2].

### Tissue Samples

The liver was obtained 1 day after OGTT under anesthesia with xylazine/ketamine (0.2 mL/100 g, intraperitoneally). Hepatectomy was performed, and the liver was perfused with cold saline and stored at − 70 °C (ROS, antioxidant species, cadmium, cytokine determination, and western blot).

#### Cadmium Concentration and Redox Balance

As previously described, cadmium concentration was determined using an inductively coupled plasma optical emission spectrometer (Mod. 730-ES, Varian Inc., USA) [2]. For the redox balance determination, 100 mg of liver tissue was homogenized in 700 mL of phosphate buffer saline (PBS) and centrifuged (2500 × g for 30 min) at 4 °C in a 17 TR microcentrifuge. Total proteins were performed using the Sedmak and Grossberg method, and all determinations were expressed in milligrams of protein. Reactive oxygen species (ROS) were measured using the 2′7′-dichlorodihydrofluorescein diacetate (DCFH-DA; Sigma Aldrich, Mexico) method in a PerkinElmer LS50-B luminescence spectrometer (Waltham, MA, USA); the results were expressed as µM/mg of protein [2, 3]. Nitrites were performed using the Griess reaction (Sigma Aldrich, Mexico) in a SmartSpec 3000 spectrophotometer (Bio-Rad, Hercules, CA, USA); the results were expressed as µM/mg of protein [2]. The concentration of malondialdehyde (MDA) and 4-hydroxyalkenals (4HDA) were quantified by colorimetric reaction using N-methyl-2-phenyl-indole diluted in a mixture of acetonitrile to methanol (3:1) and 150 µL of methanesulfonic acid or hydrochloric acid (Sigma Aldrich, Mexico) in a SmartSpec 3000 spectrophotometer (Bio-Rad, Hercules, CA, USA); the results were expressed as µM/mg of protein [2, 3].

The glutathione total, GSH, and GSSG concentrations were measured using the enzymatic recycling methods (Sigma Aldrich, Mexico) in a PerkinElmer Lambda EZ-150 spectrophotometer (Waltham, MA, USA); the results were expressed as µM/mg of protein [2, 3]. Glutathione peroxidase (GPx; nmol min^−1^/mg of protein), glutathione S-transferase (GST; U min^−1^/mg of protein), and glutathione reductase (GR; mU min^−1^/mg of protein) activities were measured by a methodology proposed by Flohé and Günzler, Habig et al., and Smith et al., respectively [2, 3]. The catalase activity (CAT; U min^−1^/mg of protein) and superoxide dismutase (SOD; U min^−1^/mg of protein) activity were quantified by Aebi and auto-oxidized pyrogallol methodologies [2, 3]. Enzymatic activities were analyzed with reactive from Sigma Aldrich, Mexico, in a PerkinElmer Lambda EZ-150 spectrophotometer (Waltham, MA, USA). Protocols are described in more detail, as we previously reported [2, 3].

#### Pro‑inflammatory and Anti‑inflammatory Cytokines

Liver tissue was homogenized with PBS, and protease inhibitors were added and centrifuged (2500 × g for 30 min) at 4 °C in a 17 TR microcentrifuge (Next Advance, Averill Park, NY). In the supernatant, IL-1β, IL-6, TNF-α, IL-10, TFG-β, and IL-1ar concentration (Merck Millipore; Toluca, Mexico) were quantified using ELISA kits following manufacturer instructions [2, 6]. Cytokine levels were normalized to total protein content and expressed as pg/mg of protein.

#### Immunoblotting

The frozen liver was homogenized in ice-cold RIPA buffer with a protease inhibitor cocktail (Complete, Roche Applied Science). After homogenization with RIPA buffer, the samples were centrifuged at 4 °C at 3000 rpm for 10 min, the supernatant was used for non-nuclear proteins, and the pellet was resuspended in nuclear extraction buffer (ab113474) with constant shaking. Finally, it was centrifuged at ~ 15,000 rpm and 4 °C for 10 min, and the supernatant was separated. The protein concentration was performed using the Sedmak and Grossberg method. SDS-PAGE was used to separate the tissue lysates after combining them with Laemmli sample buffer. The proteins were electrotransferred to a PVDF membrane after electrophoretic separation using a Mini Trans-Blot Electrophoretic Transfer Cell (Bio-Rad), blocked, and treated with the following antibodies for an overnight period at 4 °C: p-p38 (Tyr 182; sc-101759) and p-JNK (Thr 183 and Tyr 185; sc-293136), NF-κB p65 (sc-372), Nrf2 (sc-365949; Santa Cruz, CA, USA), p-Akt (Ser473; 6F5/05–1003), and p-IRS (S307; 4.2.2/05–1085) from Merck Millipore (Toluca, Mexico). After incubation with secondary antibodies conjugated with horseradish peroxidase, chemiluminescence detection was done using a chemiluminescent substrate (Immobilon Western Chemiluminescent HRP Substrate, Millipore). Digital images of the membranes were obtained with a Nine Alliance Q9 mini photodocumenter and processed with Image Lab software (Bio-Rad). The results were normalized to β-actin and Lamin A. The control samples, used as a standard for the other conditions, were given 1 as an arbitrary value.

### Statistical Analysis

The results were expressed as the mean ± SEM for all experiments. Data normality was determined by the Shapiro–Wilk test. The dose selection was analyzed by a two-way ANOVA followed by a Bonferroni test. The results were analyzed by a one-way ANOVA followed by a Tukey’s post-test. Data analysis was performed with GraphPad Prism 8 (GraphPad Software Inc., USA). (*) The significance level was set at *p* ≤ 0.05 regarding the control group. (#) The significance level was set at *p* ≤ 0.05 for the Cadmium group.

## Results

### Selection of the Curcumin Dose

To determine the optimal curcumin dose, we evaluated its effects on various parameters in rats pre-exposed to cadmium (Cd). Different curcumin doses (0, 50, 100, 250, 500, and 750 mg/kg body weight per day) were administered orally for 5 days. The highest dose (750 mg/kg/day) significantly reduced hepatic cadmium levels (Fig. S1A (*p* < 0.05), combined with Fig. S1B for ROS (*p* < 0.05)). However, all curcumin doses lowered ROS concentration compared to the Cd-exposed control group (*p* < 0.05), with no significant difference between the highest doses (500 and 750 mg/kg) and the intact control group.

Regarding insulin resistance, curcumin doses ranging from 250 to 750 mg/kg/day improved HOMA-IR (Fig. S1C, combined with Fig. S1D for hepatic insulin resistance (*p* < 0.05)). Notably, only the 250 mg/kg dose significantly improved hepatic IR. Furthermore, lower curcumin doses (50–250 mg/kg/day) appeared to be more effective in ameliorating markers of liver and kidney damage (Fig. S1E-H, specify the type of biomarkers assessed). Based on these findings, a curcumin dose of 250 mg/kg/day was chosen for further studies due to its effectiveness in reducing ROS, improving hepatic IR, and minimizing potential adverse effects on the liver and kidneys.

### Effect of Curcumin on Oxidative Stress, Antioxidant Defense, and Hepatic Cadmium

Given curcumin’s antioxidant and metal-chelating properties, we investigated its effects on these parameters in Cd-exposed rats. Sub-chronic Cd exposure for 1 month resulted in a significant increase in hepatic cadmium concentration by 8 µg/g of tissue compared to the control group. Curcumin treatment, however, reduced hepatic cadmium levels by 13% (Fig. 1A, (*p* < 0.0001)). As expected, Cd exposure significantly increased oxidative stress markers, including ROS (378%, *p* < 0.001), nitrites (90%, *p* < 0.01), MDA (72%, *p* < 0.05), and 4HDA (60%, *p* < 0.05) compared to the control group (Fig. 1B–E). Curcumin administration effectively normalized these oxidative stress markers, returning them to control group levels. Interestingly, in the intact control group (no Cd exposure), curcumin caused a 42% decrease in nitrite concentration (*p* < 0.05).

The hepatic antioxidant defense was also evaluated. Cadmium exposure led to an increase in total glutathione (81%, *p* < 0.01), GSH (59.5%, *p* < 0.05), and GSSG (125%, *p* < 0.001). Only glutathione reductase (GR) activity significantly increased (107.9%, *p* < 0.001) compared to the control group. The antioxidative index in the Cd group was reduced by 30% (*p* < 0.05). Curcumin treatment maintained elevated levels of total glutathione (44%, *p* < 0.05) and GSH (61%, *p* < 0.05) but lowered GSSG to control group levels, improving the antioxidative index by 45% (*p* < 0.05) compared to the Cd group. Regarding enzymatic activity, curcumin treatment in the Cd-exposed group maintained increased GR (79%, *p* < 0.05) and GST (65%, *p* < 0.05) activity while reducing CAT activity by 43% (*p* < 0.05) and restoring GPx activity to control levels. In the intact control group, curcumin increased GR and GST activity (*p* < 0.05) but decreased CAT activity (*p* < 0.05; Table 1).

**Table 1:** Effect of curcumin administration on hepatic antioxidant defense after cadmium exposure

	Control	Curcumin	Cadmium	Cd + Cur
Total glutathione (µM/mg of protein)	18.9 ± 2.5	20.6 ± 3.6	34.3 ± 3.3*	27.3 ± 2.2*#
GSH (µM/mg of protein)	12.6 ± 1.8	13.9 ± 1.2	20.1 ± 2.2*	20.3 ± 1.4*
GSSG (µM/mg of protein)	6.3 ± 0.6	6.7 ± 0.7	14.2 ± 1.4*	7 ± 0.8#
2GSH/GSSG	4.0 ± 0.5	4.1 ± 0.6	2.8 ± 0.1*	5.8 ± 0.3*#
GPx nmol min^−1^/mg of prot	2.3 ± 0.1	3.6 ± 0.1	3.8 ± 0.4	2.6 ± 0.4#
GR mU min^−1^/mg of prot	1147.7 ± 170	2092 ± 222*	2386.5 ± 260*	2053 ± 117*
GST U min^−1^/mg of prot	658.2 ± 92	1332 ± 124*	733.3 ± 128	1087 ± 76*
SOD U min^−1^/mg of prot	7.6 ± 0.9	7.9 ± 0.6	10.9 ± 0.5	10.7 ± 0.5
CAT U min^−1^/mg of prot	19.5 ± 1.8	7 ± 0.5*	24.8 ± 2.1	11.1 ± 1.6*#

The results shown are the average of five different experiments ± SEM. A one-way ANOVA test followed by a Tukey’s post-test with a significance level set at *p* ≤ 0.05 was performed. *A significant difference regarding the control group. #A significant difference regarding the Cd group. *GSH*, reduced glutathione; *GSSG*, oxidized glutathione; *GPx*, glutathione peroxidase; *GR*, glutathione reductase; *GTS*, glutathione S-transferase; *SOD*, superoxide dismutase; *CAT*, catalase

### Effect of Curcumin on Hepatic Inflammation and Anti-inflammatory Response

**Fig. 2:**
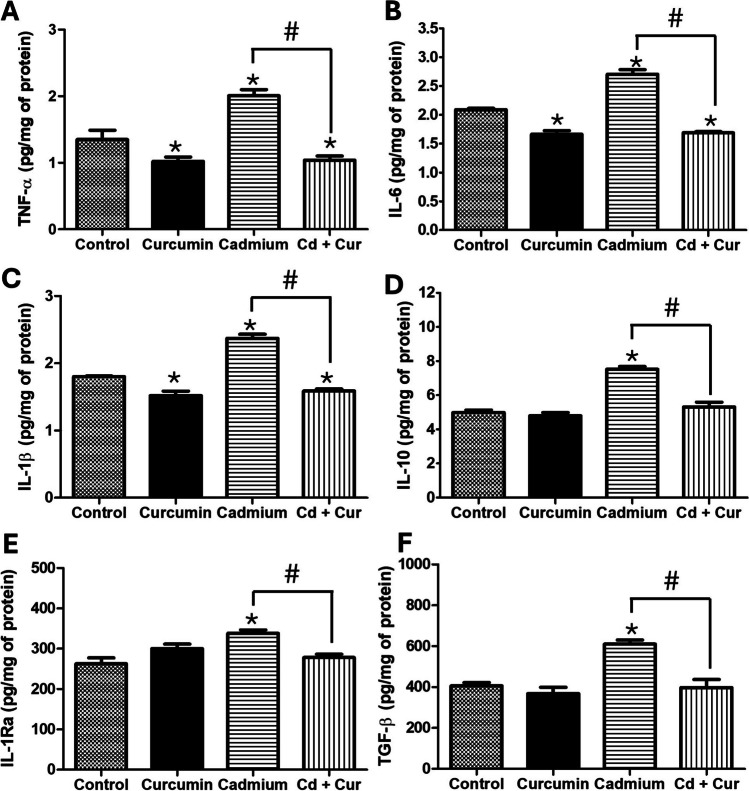
Effect of curcumin administration on hepatic inflammation and anti-inflammation after cadmium exposure. **A** TNF-α; **B** IL-1β; **C** IL-6; **D** IL-10; **E** IL-1Ra; **F** TGF-β. The results shown are the average of five different experiments ± SEM. The symbol “*” indicates a significant difference regarding the control group (*p* ≤ 0.05); the symbol “#” indicates a significant difference regarding the cadmium group (*p* ≤ 0.05), by a one-way ANOVA followed by a Tukey’s post-test

Since oxidative stress is closely linked to inflammation, we evaluated the effect of curcumin on liver cytokine levels in Cd-exposed rats. Cadmium exposure significantly increased the concentration of both pro-inflammatory cytokines TNF-α, IL-6, and IL-1β (48%, 29%, and 31% (*p* < 0.05)) and anti-inflammatory cytokines IL-10, IL-1Ra, and TGF-β (51%, 22%, and 50% (*p* < 0.05)) compared to the control group. Curcumin administration effectively reduced the concentration of all these cytokines, with reductions ranging from 17.7 to 48.3% (Fig. 2, *p* < 0.05). Interestingly, curcumin treatment normalized the levels of anti-inflammatory cytokines in the Cd-exposed group, but it also reduced the levels of pro-inflammatory cytokines below those observed in the control group. This suggests a potential curcumin-mediated anti-inflammatory response.

**Fig. 3:**
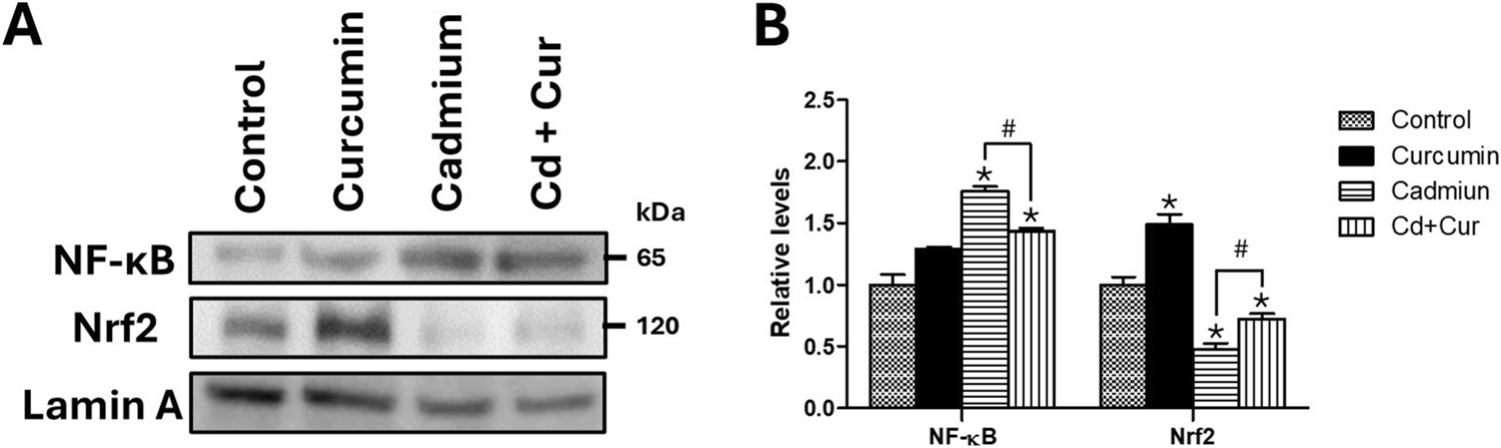
Effect of curcumin administration on hepatic expression of Nrf2 and NF-κB after cadmium exposure. **A** Western blot analysis of protein Nrf2 and NF-κB expression of the nuclear extracts of the liver after curcumin administration. **B** Densitometric analysis of Nrf2 and NF-κB expression. The results shown are the average of five different experiments ± SEM. The symbol “*” indicates a significant difference regarding the control group (*p* ≤ 0.05); the symbol “#” indicates a significant difference regarding the cadmium group (*p* ≤ 0.05), by a one-way ANOVA followed by a Tukey’s post-test

To further explore the mechanisms underlying these findings, we analyzed the hepatic expression of Nrf2 and NF-κB, two key transcription factors involved in the regulation of inflammation and antioxidant defense. Chronic Cd exposure led to a 75% increase in hepatic nuclear NF-κB expression (*p* < 0.01), which was significantly reduced by 18% following curcumin treatment (*p* < 0.05; Fig. 3B). Notably, curcumin administration caused a slight increase in NF-κB expression without inducing inflammatory cytokine expression. In addition, Nrf2 expression was significantly decreased by 53% in the Cd-exposed group (*p* < 0.05). Curcumin treatment partially restored Nrf2 expression, resulting in a 47% increase compared to the Cd group (*p* < 0.05), although it remained lower than control levels. Interestingly, curcumin administration in the intact control group further increased Nrf2 expression by 48% (Fig. 3B, p < 0.05).

### Effect of Curcumin on Hepatic Insulin Signaling Pathways

**Fig. 4:**
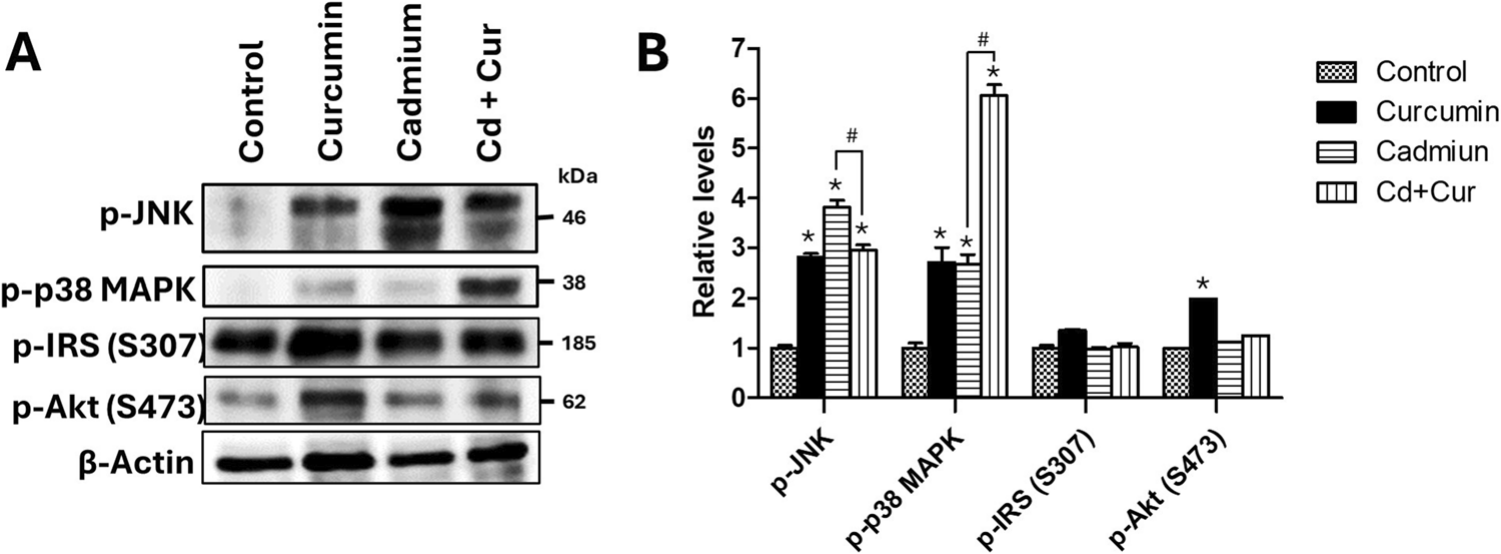
Effect of curcumin administration on hepatic p-JNK, p-p38, p-IRS, and p-Akt after cadmium exposure. **A** Western blot analysis of the expression of the protein p-JNK, p-p38 MAPK, p-IRS (S307), and p-Akt (S473) cytoplasmic extracts of the liver after administration of curcumin. **B** Densitometric analysis of p-JNK, p-p38 MAPK, p-IRS (S307), and p-Akt (S473). The results shown are the average of five different experiments ± SEM. The symbol “*” indicates a significant difference regarding the control group (*p* ≤ 0.05); the symbol “#” indicates a significant difference regarding the cadmium group (*p* ≤ 0.05), by a one-way ANOVA followed by a Tukey’s post-test

Insulin resistance is a complex process influenced by oxidative stress, inflammation, and MAPK pathway. The MAPK pathways, along with IRS and Akt, are key components of the insulin signaling cascade that regulates glucose metabolism in cells. To investigate the effects of curcumin on these pathways, we analyzed the expression of phosphorylated JNK (p-JNK) and p38 (p-p38), markers of MAPK activation, as well as phosphorylated IRS (p-IRS) and Akt (p-Akt). Cadmium exposure significantly increased the phosphorylation of both JNK and p38 compared to the control group (381% and 167%, *p* < 0.001). Interestingly, curcumin treatment effectively reduced p-JNK phosphorylation to 22% of control levels, *p* < 0.05. However, curcumin administration unexpectedly led to a further increase in p-p38 phosphorylation (126%, *p* < 0.001) compared to the Cd-exposed group. Notably, curcumin itself caused an increase in both p-JNK and p38 phosphorylation in the intact control group (Fig. 4B).

We also analyzed p-IRS and p-Akt expression, as these proteins play a crucial role in insulin signaling downstream of the insulin receptor. Neither curcumin treatment nor cadmium exposure resulted in significant changes in p-IRS or p-Akt levels compared to the control group. However, curcumin administration in the control group did lead to a slight increase in total Akt expression (16%, *p* < 0.05).

### Effect of Curcumin on Oral Glucose Tolerance and Insulin Resistance

Cadmium exposure significantly impaired oral glucose tolerance, as indicated by elevated blood glucose levels at all time points measured (basal, 86%; 30 min, 91%; 60 min, 92%; and 90 min, 89%; *p* < 0.01) compared to the control group. The overall glucose response, reflected by the area under the curve (AUC), was also significantly increased by 90% (*p* < 0.01) following Cd exposure. Furthermore, Cd exposure increased serum insulin concentrations at all time points (basal, 85%; 30 min, 68%; 60 min, 67%; and 90 min, 50%; *p* < 0.05) compared to control, resulting in a 51% increase in the insulin AUC. These changes were reflected in elevated HOMA-IR (60%, *p* < 0.05) and LIRI (66%, *p* < 0.05) indices, indicative of insulin resistance (Table 2).

**Table 2:** Effect of curcumin administration on oral glucose tolerance, insulin response, and insulin resistance indices after cadmium exposure

	Control	Curcumin	Cadmium	Cd + Cur
Fasting glucose (mg/dL)	93 ± 3.8	95 ± 2.6	106.6 ± 3.6*	92 ± 4.1#
Glucose 30′ (mg/dL)	114.4 ± 5.1	120 ± 2.8	148.5 ± 3.4*	135.5 ± 3.1*#
Glucose 60′ (mg/dL)	89.6 ± 4	131 ± 2.8*	175 ± 6*	159.3 ± 5.2*#
Glucose 90′ (mg/dL)	97 ± 5	120 ± 5.7*	169 ± 4.6*	155 ± 8.6*#
Glucose AUC	7994 ± 705	9953 ± 691	14,028 ± 678*	12,710 ± 859*
Fasting insulin (µUI/mL)	12.1 ± 1.6	12.06 ± 1.9	22.1 ± 1.1*	19 ± 2.2
Insulin 30′ (µUI/mL)	13.5 ± 2.1	15.9 ± 2.2	29.5 ± 2.6*	20.2 ± 2.1*#
Insulin 60′ (µUI/mL)	12.6 ± 2.3	14.0 ± 1.1	34 ± 3.2*	23 ± 2.9*#
Insulin 90′ (µUI/mL)	9.6 ± 1.5	11.1 ± 1.7	36 ± 2.4*	18 ± 2.3*#
Insulin AUC	1102 ± 57	1221 ± 64	3199 ± 49*	1655 ± 120*#
HOMA-IR	0.45 ± 0.01	0.48 ± 0.02	1.0 ± 0.04*	0.6 ± 0.02*#
LIRI	0.18 ± 0.08	0.29 ± 0.04	0.72 ± 0.08*	0.48 ± 0.01*#

The results shown are the average of five different experiments ± SEM. A one-way ANOVA test followed by a Tukey’s post-test with a significance level set at *p* ≤ 0.05 was performed. *A significant difference regarding the control group. #A significant difference regarding the Cd group. *AUC*, area under the curve; *HOMA-IR*, homeostasis model assessment insulin resistance; *LIRI*, liver insulin resistance index

Curcumin administration partially improved glucose tolerance in Cd-exposed rats. Blood glucose levels were moderately reduced at all time points (basal, 13%; 30 min, 8.7%; 60 min, 9%; and 90 min, 8.2%; *p* < 0.05) compared to the Cd group, with a corresponding decrease in the overall glucose response (AUC, 9.5% (*p* < 0.05)). Curcumin treatment also improved insulin response, reflected by reduced insulin concentrations at all time points (basal, 14%; 30 min, 31%; 60 min, 32%; and 90 min, 50%; *p* < 0.05) and a decrease in the insulin AUC (48%, *p* < 0.05), reducing HOMA-IR and LIRI indices, although they did not reach control levels. Interestingly, curcumin administration in the intact control group (no Cd exposure) did not affect insulin secretion or resistance indices but slightly impaired glucose tolerance during the latter phase of the test (60 and 90 min) (Table 2).

## Discussion

Building on the finding that curcumin at 250 mg/kg/day effectively reduced hepatic cadmium concentration, improved insulin resistance, and minimized organ damage (Fig. S1), we investigated whether this dose could counteract the molecular pathways altered by Cd exposure. Our results provide evidence supporting curcumin’s potential to mitigate Cd-induced hepatic insulin resistance. Curcumin’s well-documented metabolic benefits in humans align with our findings. Studies have shown its effectiveness in improving β-cell function and reducing insulin resistance at doses ranging from 1.5 g/day for 9 months [15] to 180 mg/day [16]. Additionally, curcumin’s ability to reduce inflammation and oxidative stress was observed in studies using dosages of 360 mg three times daily [17], which is likely relevant to the mechanisms by which it combats cadmium’s effects. Similarly, animal studies using curcumin at doses of 0.2 g/kg for 6 weeks and 30–90 mg/kg daily reported positive effects on regulating lipid metabolism and blood sugar control [18].

Curcumin’s mechanism of action in metabolic disorders involves multiple pathways, including PI3K-Akt, MAPK, peroxisome proliferator-activated receptor gamma (PPAR-γ), cyclooxygenases, and SMAD proteins [19–21]. Also, curcumin can reduce the expression or inhibit the activity of gluconeogenic enzymes such as phosphoenol pyruvate kinase, glucose-6 phosphatase, and α-glucosidase. This highlights its potential to create a more favorable cellular environment for insulin signaling and glucose metabolism in Cd-exposed rats. Importantly, the optimal curcumin dose depends on various factors, including disease severity, comorbidities, and administration methods. Previous studies have established that low-dose cadmium exposure (NOAEL and LOAEL doses) can lead to metabolic disorders [2, 5–7, 22], which opens new avenues for investigating curcumin as a therapeutic strategy for Cd-induced metabolic complications.

Curcumin’s mechanism of action against cadmium’s effects likely involves a combination of its antioxidant and metal-chelating properties. While the keto form, prevalent in physiological environments, exhibits weaker metal chelation than the enolic form, our findings suggest it may still modestly reduce Cd accumulation (Fig. 1A). This could be through direct chelation or indirect mechanisms limiting Cd uptake and, in consequence, its toxic effect. However, we observed an elevation in glutathione (GSH) levels in the Cd group (Table 1), and the overall redox state remained oxidative (Fig. 1). This highlights the complex interplay between Cd exposure, antioxidant defense, and free radical generation. Our previous works have shown that the severity of cadmium’s oxidative effects depends on factors like dose and duration of exposure [2, 6]. Although cadmium itself does not directly generate free radicals, it can disrupt cellular homeostasis, increasing ROS production and lipid peroxidation (Fig. 1). Cells employ cysteine-rich molecules like metallothionein and GSH to neutralize Cd and free radicals, forming complexes for excretion, eliminating them from the liver, and detoxifying and protecting the tissue from cadmium’s harmful effects [23].

However, studies using high or acute cadmium doses, often exceeding LOAEL or NOAEL limits, have not always shown a consistent protective effect of curcumin on GSH levels. Tarasub et al. co-treated rats with cadmium acetate (200 mg/kg) and curcumin (250 mg/kg), finding that curcumin could not prevent Cd-induced oxidative damage. These discrepancies suggest that Cd dose and exposure duration may influence curcumin’s effectiveness [24].

Curcumin treatment effectively restored the redox balance in the liver of Cd-exposed rats, reducing markers of oxidative stress (GSSG, ROS, MDA, 4HDA, nitrite) to levels comparable to the control group (Fig. 1B–D). Curcumin’s antioxidant properties likely contribute to these protective effects in Cd-exposed rats. Its ability to directly scavenge free radicals, including ROS and reactive nitrogen species, provides a first line of defense through the phenolic hydroxyl group, which is an electron-donating group and is the main contributor to its antioxidant activity [17, 18]. Research studies have shown that curcumin (50 or 250 mg/kg) prevents lipid peroxidation in rats with administered cadmium chloride or cadmium acetate. The same effects have been observed in mice preconditioned with curcumin and intoxicated with cadmium [25, 26]. This aligns with previous findings demonstrating curcumin’s ability to prevent lipid peroxidation in Cd-treated animals. Considering the potent antioxidant activity, curcumin administration can restore abnormal alternations induced by cadmium and other external factors, repressing transcription factors related to oxidation. These effects help reduce oxidative stress and lower the risk of chronic diseases.

Notably, curcumin treatment also enhanced the activity of antioxidant enzymes (glutathione enzymes) while reducing CAT activity in both Cd-exposed and control rats (Table 1). Our results evidence that curcumin activates phase II detoxifying enzymes. Curcumin enhances the activity of phase II detoxifying enzymes like those in the glutathione system, CAT, and SOD by activating the Nrf2 pathway [15–17]. Mechanistically, curcumin acts through the Nrf2/Kelch-like ECH-associated protein 1/antioxidant response elements (Nrf2/Keap1/ARE) pathway. Curcumin modifies Keap1’s cysteine residues, leading to a conformational change that liberates Nrf2. Nrf2 then translocates to the nucleus and binds to ARE in DNA, initiating the transcription of antioxidant genes encoding enzymes like SOD, CAT, and glutathione enzymes [14, 17, 27]. As observed in our study (Fig. 4), curcumin treatment increased Nrf2 levels in both Cd-exposed and control groups, potentially reinforcing the antioxidant defense system. In contrast, Nrf2 expression was diminished in the Cd-exposed group without curcumin treatment. This finding aligns with previous reports suggesting that cadmium exposure can impair Nrf2 activity and lead to the activation of NF-κB, inflammasome NLRP3, and MAPK signaling pathways [28]. These pathways are known to contribute to metabolic disorders, steatosis, and liver diseases [28, 29]. By restoring Nrf2 activity, curcumin treatment may help mitigate these downstream effects of Cd exposure.

Our findings suggest that cadmium exposure disrupts the interplay between Nrf2 and NF-κB signaling pathways, potentially contributing to the observed oxidative stress and inflammation. The increased nuclear localization of NF-κB in the cadmium group (Fig. 4) likely led to Nrf2 inactivation by preventing its heterodimerization, which could disrupt the transcription of antioxidant genes regulated by Nrf2 [27]. Previous studies support this link, demonstrating that Nrf2 inhibition can be associated with enhanced NF-κB activation [28, 30, 31]. Likewise, NF-κB, a redox-sensitive transcription factor, also plays a key role in inflammatory responses. ROS and reactive nitrogen species (RSN) generated by cadmium exposure can activate NF-κB, leading to the production of pro-inflammatory cytokines [27, 28]. Our data confirm this, showing an increase in pro-inflammatory cytokines (TNF-α, IL-6, IL-1β) in the cadmium group (Fig. 2). Interestingly, the cadmium group also exhibited an upregulation of anti-inflammatory cytokines (IL-10, IL-1Ra, TGF-β), suggesting an attempt to regulate the inflammatory response (Fig. 2). The cellular sources of these cytokines likely involve hepatocytes, M1 macrophages, and T-helper lymphocytes. Hepatocytes and M1 macrophages are known to produce and secrete inflammatory cytokines via NF-κB signaling [32, 33]. T-helper cells (Th1, Th2, Th17, and regulatory T cells) are a primary source of IL-10 in humans and mice, while hepatocytes produce IL-1Ra and TGF-β primarily through the MAPK/AP-1 pathway, particularly via p38 and JNK activation [34, 35]. Notably, our data also showed increased p38 and JNK-MAPK phosphorylation in the cadmium group (Fig. 3), further supporting the activation of this inflammatory signaling cascade.

Curcumin’s anti-inflammatory properties likely contribute to its protective effects against cadmium exposure. Curcumin can inhibit the activity of enzymes like cyclooxygenase and lipoxygenase, which are involved in the production of inflammatory mediators. Additionally, it can block the activation of NF-κB and AP-1, key transcription factors that regulate the expression of pro-inflammatory cytokines like IL-6, TNF-α, and MCP-1, maintaining the S-nitrosylation of the inhibitor of NF-κB kinase subunit β (IKKβ), which represses the phosphorylation of IκB and the activation of NF-κB. Curcumin derivatives contain nonsteroidal anti-inflammatory components that block the phosphorylation of IκB-α and suppress the activation of NF-κB and IκB-α [14, 17]. Studies support this link, showing that curcumin supplementation can reduce TNF-α and IL-6 levels in Cd-exposed rats [25, 36]. Our findings demonstrate that curcumin treatment reduced nuclear NF-κB levels in the cadmium group (Fig. 4). This likely led to the normalization of hepatic cytokine levels, with both pro-inflammatory (TNF-α, IL-6, IL-1β) and anti-inflammatory (IL-10, IL-1Ra, TGF-β) cytokines returning to basal levels or even falling below control group levels in both Cd-exposed and non-exposed groups (Fig. 2). This suggests a potential dual regulatory effect of curcumin on cytokine expression.

Curcumin can also modulate the MAPK pathway, a signaling cascade involved in inflammation. While most studies report curcumin-mediated inhibition of MAPKs, our data showed an increase in p38 phosphorylation in both Cd-exposed and non-exposed groups after curcumin treatment (Fig. 3). This finding suggests a context-dependent effect of curcumin on p38, where upregulation might be associated with its beneficial actions in some scenarios [21, 37]. Different studies have exposed this curcumin effect with beneficial results, such as suppression of tumors, glucose uptake, and ameliorated inflammation in various tissues [20, 38], which at least in part also depends on p38 [37]. Further research is needed to elucidate the specific role of p38 upregulation by curcumin in our model. However, most studies mentioned inhibition or decreased MAPK activity and expression after curcumin treatment. In contrast, curcumin treatment reduced JNK phosphorylation in the Cd group but increased it in the non-exposed group (Fig. 3). This suggests that JNK activation may be more tightly linked to the oxidative stress and NF-κB signaling induced by Cd exposure. Collectively, our results suggest that curcumin’s anti-inflammatory effects involve modulation of both NF-κB and MAPK signaling pathways, with the specific effects on MAPKs potentially depending on the cellular context.

Our findings suggest that curcumin’s protective effects against cadmium-induced insulin resistance may be mediated, at least partly, through its ability to modulate inflammation and oxidative stress rather than directly through the IRS-Akt pathway. Inflammatory cytokine regulation and amelioration of the hepatic redox environment with a curcumin treatment positively impacted the glucose homeostasis and insulin signaling pathways. While previous studies by our group have shown that chronic Cd exposure disrupts IRS-Akt signaling and leads to IR [5, 6], curcumin treatment in the present study did not alter IRS-1 or Akt phosphorylation in the Cd group (Fig. 3). Despite this, curcumin effectively improved postprandial hyperglycemia, hyperinsulinemia, and hepatic IR (Table 2), suggesting that Cd-induced IR in our model may be primarily driven by the inflammatory and oxidative stress environment rather than solely by alterations in the IRS-Akt pathway. This concept is supported by evidence that increased JNK and p38 MAPK signaling, often triggered by Cd exposure, can impair insulin receptor and substrate phosphorylation, leading to IR [2, 5, 6]. Our data showed a reduction in JNK phosphorylation with curcumin treatment in the cadmium group (Fig. 3), potentially contributing to the observed improvement in insulin sensitivity.

Previously, we demonstrated that chronic and subacute Cd exposures to NOAEL and LOAEL doses induce IR through the IRS-Akt pathway [2, 5, 6]. Curcumin’s ability to improve insulin signaling has been documented in studies of diabetes, obesity, and IR models, where it often enhances the IRS-Akt pathway [15, 18, 19]. Interestingly, curcumin treatment in the non-exposed group significantly increased IRS-1 phosphorylation compared to Akt (Fig. 3). However, these rats did not exhibit IR. This suggests that in healthy livers, curcumin may modulate the IRS-1/PI3K/Akt pathway to activate survival pathways and potentially enhance metabolic efficiency [19]. Future studies are needed to explore these possibilities further.

In summary, sub-chronic exposure to the low-observable adverse effect level (LOAEL) dose of cadmium resulted in hepatic insulin resistance associated with several factors, such as Cd accumulation in the liver, increased oxidative stress, inflammation, and disrupted expression of key signaling molecules (NF-κB, Nrf2, JNK, and p38 MAPKs). Our study investigated the potential protective effects of curcumin at a dose of 250 mg/kg/day against these cadmium-induced changes. While curcumin exhibited modest cadmium-chelating activity, its primary benefit appeared to be its potent antioxidant properties. Curcumin treatment for 5 days effectively restored the redox balance in the liver (reduced oxidative stress). Curcumin also displayed anti-inflammatory properties, normalizing the levels of both pro-inflammatory and anti-inflammatory cytokines. Additionally, it helped restore the balance between NF-κB and Nrf2 expression, potentially modulating the MAPK pathway. These combined effects likely contributed to the observed improvement in hepatic insulin resistance, glycemia, and insulinemia after curcumin treatment. In conclusion, our findings suggest that Cd exposure disrupts key signaling pathways in the liver, leading to IR via oxidative stress, inflammation, and altered MAPK activity. Curcumin treatment appears to counteract these effects by restoring redox balance, modulating inflammatory responses, and potentially influencing the interplay between NF-κB and Nrf2 signaling.

## Supplementary Information

Below is the link to the electronic supplementary material.

Supplementary file1. Figure S1. Effect of different doses of curcumin on ROS, cadmium, IR indices, and markers of kidney and liver damage after cadmium exposure. Results are the mean average of 5 separate experimental animals per group ± SEM. (*) indicates a significant difference regarding the control group (p ≤ 0.05); (#) indicates a significant difference regarding the cadmium group (p ≤ 0.05), by a two-way ANOVA followed by a Bonferroni test (JPG 1093 KB)

## Data Availability

No datasets were generated or analysed during the current study.
