# Supplementary figures and images for: Curcumin Treatment Ameliorates Hepatic Insulin Resistance Induced by Sub-chronic Oral Exposure to Cadmium LOAEL Dose via NF-κB and Nrf2 Pathways
Source: Biol Trace Elem Res. 2024 Aug 6;203(4):2382–93. doi: 10.1007/s12011-024-04314-1 (PMC11919948; doi:10.1007/s12011-024-04314-1)

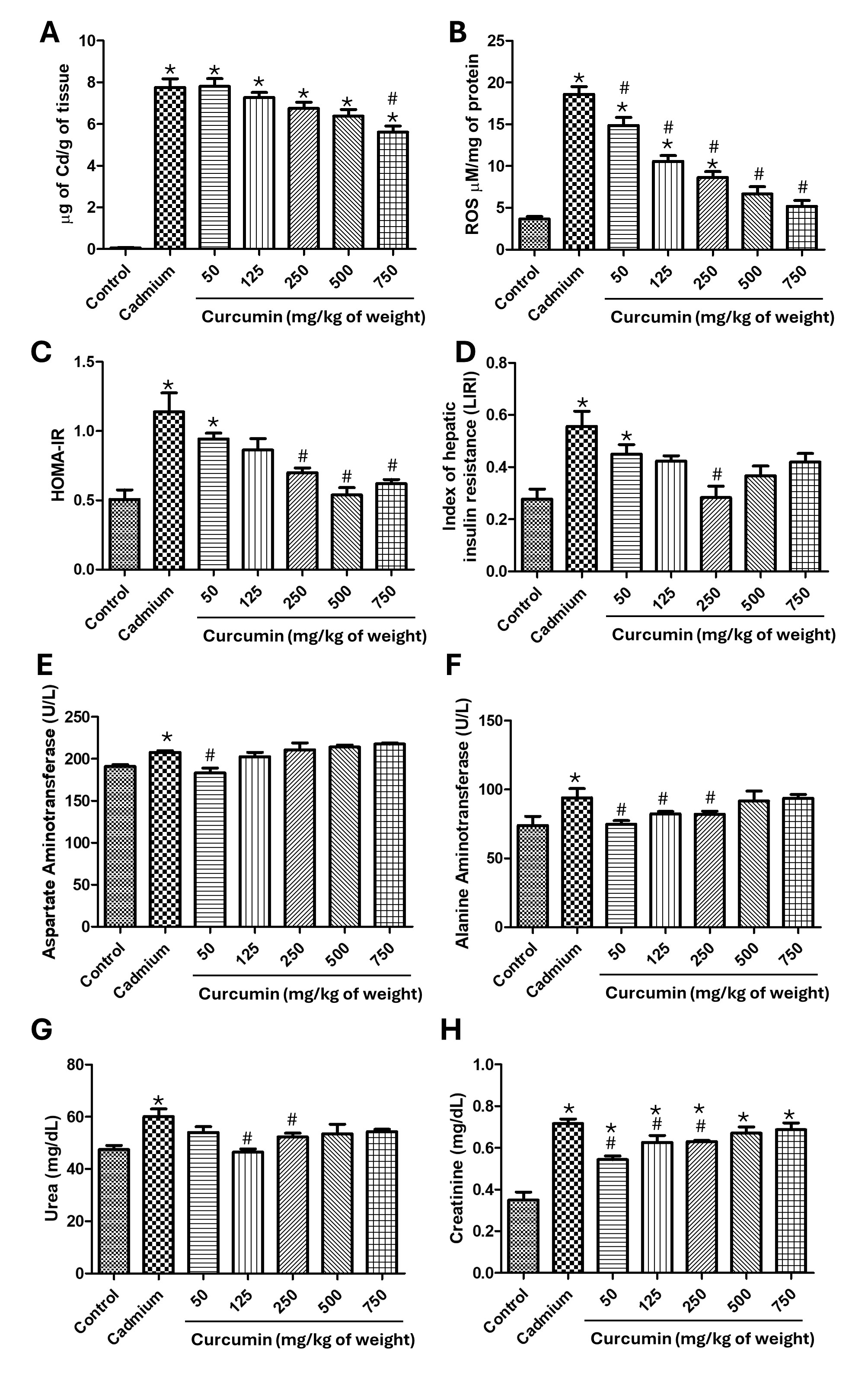

Supplement: Supplementary file 1 — Supplementary file1. Figure S1. Effect of different doses of curcumin on ROS, cadmium, IR indices, and markers of kidney and liver damage after cadmium exposure. Results are the mean average of 5 separate experimental animals per group ± SEM. (*) indicates a significant difference regarding the control group (p ≤ 0.05); (#) indicates a significant difference regarding the cadmium group (p ≤ 0.05), by a two-way ANOVA followed by a Bonferroni test (JPG 1093 KB) [file 12011_2024_4314_MOESM1_ESM.jpg]
